# Utilizing systematic Mendelian randomization to identify potential therapeutic targets for mania

**DOI:** 10.3389/fpsyt.2024.1375209

**Published:** 2024-03-05

**Authors:** Fang-Biao Xu, Sen Hu, Jing-Jing Wang, Xin-Zhi Wang

**Affiliations:** ^1^ Department of Encephalopathy, The First Affiliated Hospital of Henan University of Chinese Medicine, Zhengzhou, China; ^2^ The First Clinical Medical College, Henan University of Chinese Medicine, Zhengzhou, China; ^3^ Department of Medical Records, Zhengzhou University People’s Hospital, Henan Provincial People’s Hospital, Zhengzhou, Henan, China; ^4^ Neurology Department, The First Affiliated Hospital of Zhengzhou University, Zhengzhou, China

**Keywords:** mania, Mendelian randomization, drug targets, bioinformatic analysis, phenylalanine

## Abstract

**Background:**

Mania has caused incalculable economic losses for patients, their families, and even society, but there is currently no effective treatment plan for this disease without side effects.

**Methods:**

Using bioinformatics and Mendelian randomization methods, potential drug target genes and key substances associated with mania were explored at the mRNA level. We used the chip expression profile from the GEO database to screen differential genes and used the eQTL and mania GWAS data from the IEU database for two-sample Mendelian randomization (MR) to determine core genes by colocalization. Next, we utilized bioinformatics analysis to identify key substances involved in the mechanism of action and determined related gene targets as drug targets.

**Results:**

After differential expression analysis and MR, a causal relationship between the expression of 46 genes and mania was found. Colocalization analysis yielded six core genes. Five key substances were identified via enrichment analysis, immune-related analysis, and single-gene GSVA analysis of the core genes. MR revealed phenylalanine to be the only key substance that has a unidirectional causal relationship with mania. In the end, SBNO2, PBX2, RAMP3, and QPCT, which are significantly associated with the phenylalanine metabolism pathway, were identified as drug target genes.

**Conclusion:**

SBNO2, PBX2, RAMP3, and QPCT could serve as potential target genes for mania treatment and deserve further basic and clinical research. Medicinal target genes regulate the phenylalanine metabolism pathway to achieve the treatment of mania. Phenylalanine is an important intermediate substance in the treatment of mania that is regulated by drug target genes.

## 1 Introduction

As the main clinical phenomenon, mania is characterized primarily by elevated mood and irritability, accompanied by heightened energy, increased speech, and increased activity. In severe cases, there are symptoms of psychosis, including hallucinations, delusions, and anxiety. Manic episodes must last for more than a week and usually present as a recurring illness. After each episode, patients enter a remission period during which their mental state returns to normal. Most patients have a tendency for recurring episodes (1). Long-term observation of mood disorders has revealed that it is rare for patients to have manic or hypomanic episodes only, and these patients’ family histories, premorbid personalities, biological characteristics, principles of treatment, and prognoses are similar to those of patients with bipolar disorder who have both manic and depressive episodes. This finding also demonstrates the importance of the ICD-11. There were a total of 8 diagnoses associated with mania, 2 of which were primary psychotic disorders with manic symptoms and secondary mood syndromes accompanying manic symptoms. The other 6 all had bipolar disorder (BD), and clinicians often use manic behavior as a diagnostic criterion for BD.

At present, the pathophysiology and neurobiological mechanisms of mania have not been fully elucidated. A large amount of research has suggested that this phenomenon may be related to neurotransmitter imbalance, neuroinflammation, circuity biology, hormones, and environmental factors. First, an imbalance in neurotransmitter levels is considered a key element in creating this condition. Specific neurotransmitters, such as dopamine, serotonin, and norepinephrine, are believed to play a major role in the manic phase (2) because during this stage, the brain may produce too much dopamine, which could lead to excessive excitement and overactivity. Second, there is currently evidence suggesting that neuroinflammation may play a crucial role in the development of bipolar disorder. This has gained increasing support, as many studies have shown that certain inflammatory markers in the human body are elevated in patients with mania (3) and affect cognitive function to a certain extent. In addition, abnormalities in neural circuitry are also viewed as a possible cause. This mainly involves structural and functional abnormalities in the frontal lobe. Research shows that during mania, the activities of these areas change significantly (4). Hormones are also thought to influence the development of mania. Several studies have shown that estrogens and androgens may play key roles in women and men experiencing bipolar disorder (5). Environmental factors, such as stress, are also considered factors that may lead to mania. The possible mechanisms of mania are multifactorial, and additional research is needed to determine more specific mechanisms to better understand mania.

Mania affects more than 1% of the global population (6), and the suicide rate among mania patients is relatively high among psychiatric patients. This is because when patients are in a manic state, they usually feel good about themselves, have no self-awareness of their illnesses, have heightened emotions, have racing thoughts, increased volition and actions, weak impulse control, and impaired judgement, resulting in irrational behaviour. Mania tends to recur, leading to chronic disease, personality changes, and impairments in social functioning, thus harming the individual’s and their family’s economic and personal safety and, in some cases, the public’s safety. Therefore, it is highly important to find more effective treatment plans.

Bioinformatics is the discipline of collecting, processing, storing, disseminating, analysing, and interpreting various aspects of biological information. It is a new discipline formed with the rapid development of life sciences and computer science (7). Comprehensive use of biology, computer science, and information technology has revealed the biological mysteries associated with a plethora of complex biological data (8).

The binding site of the drug and the biological macromolecules of the organism are the drug targets. Drug action targets include receptors, enzymes, ion channels, transporters, the immune system, genes, etc. Mendelian randomization aims to use genetic variation to evaluate whether the exposure–outcome relationship might be causal (9). Because genetic mutations are randomly assigned at conception and do not randomly change over time, they can be used as a tool to evaluate the causal effects of randomly assigned exposure on the outcome. The definition of drug targets spans transcription, translation, modification, tissues, systems, and phenotypes. Therefore, Mendelian randomization of drug targets can be simply considered Mendelian randomization involving receptors, enzymes, ion channels, transporters, the immune system, genes, etc (10).

**Figure 1:**
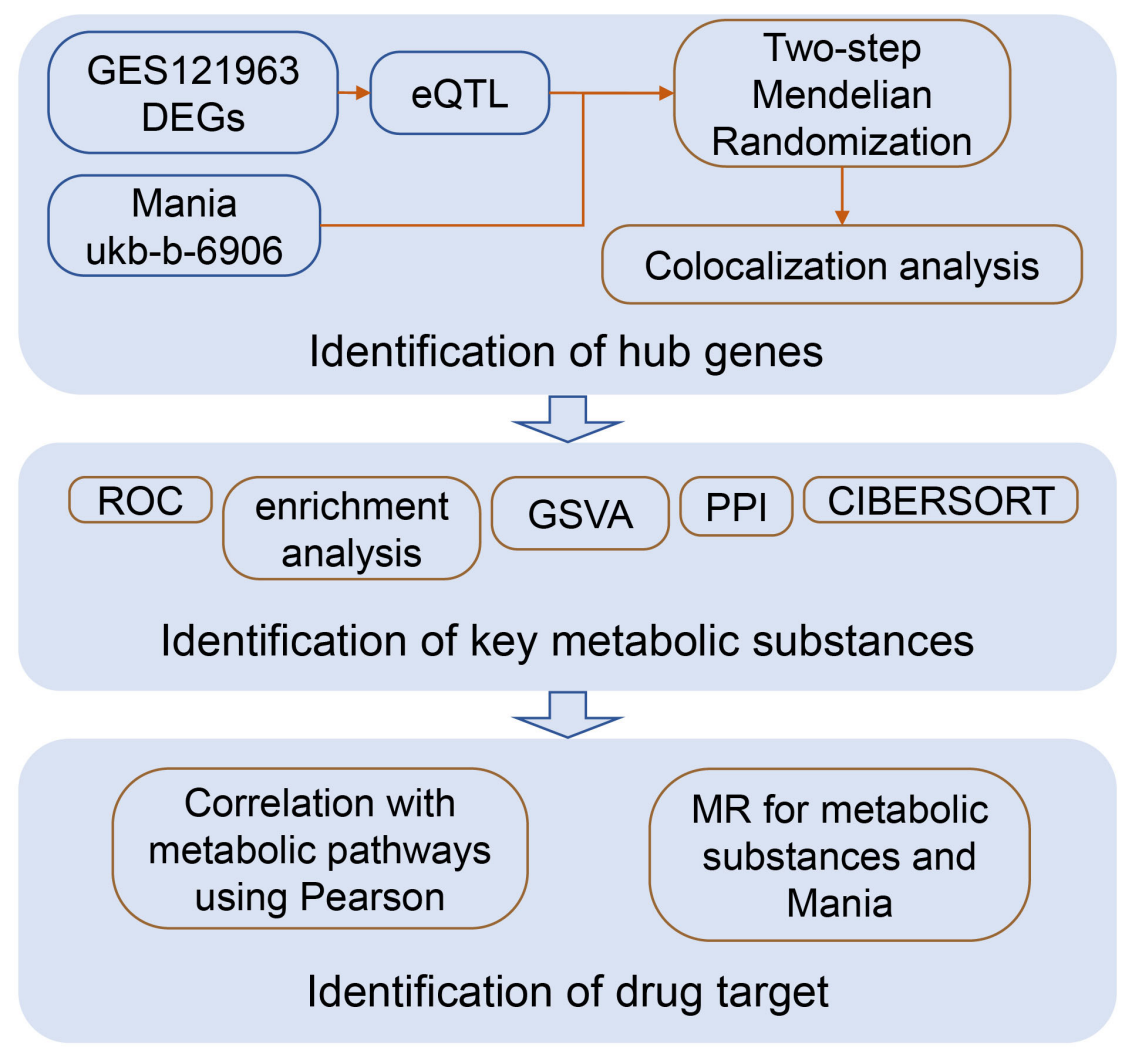
Flowchart of the study.

This research integrates bioinformatics and Mendelian randomization, mining potential drug target genes for mania at the mRNA level, drug target-related molecular mechanisms, and the material basis with causal relationships. A conceptual diagram of this study is shown in **Figure 1**.

## 2 Methods

### 2.1 Data preparation

The chip expression spectra were obtained from the Gene Expression Omnibus (GEO) database, and we searched for the term “manic episode”. Using GPL17586 human peripheral blood samples, the GES121963 (https://www.ncbi.nlm.nih.gov/geo/query/acc.cgi?acc=GSE121963) included gene expression data on the manic episodes and remission periods of 6 patients. Because of tissue-specific expression, we chose the eQTLGen database (11) (https://www.eqtlgen.org/) derived from blood samples as the exposure dataset and the most sample size GWAS dataset of mania from UKB database of the European population (https://www.ukbiobank.ac.uk/) as the outcome. The key substance data were retrieved from the IEU database (https://gwas.mrcieu.ac.uk/). All our data were retrieved and obtained from the IEU. A detailed introduction to the data can be found in **Table 1**.

**Table 1:** Summary of the data used for the MR analysis.

Name	Datasets	Sample size	Count	Year
**eQTL**	eqtl-a	477	19942 genes	2019
**mania**	ukb-b-6906	462933	9851867 SNPs	2018
**isoleucine**	met-d-Ile	115075	12321875 SNPs	2020
**leucine**	met-d-Leu	115074	12321875 SNPs	2020
**phenylalanine**	met-d-Phe	115025	12321875 SNPs	2020
**valine**	met-d-Val	115048	12321875 SNPs	2020
**vitamin B6**	ukb-b-7864	64979	9851867 SNPs	2018

### 2.2 Obtaining mania-associated genes with causal relationships

First, we used the more rigorous voom pipeline (12) in the limma package for differential analysis. Next, we screened significant and independent SNPs for each differential gene as instrumental variables through the eQTLGen database. We selected genome-wide significant single nucleotide polymorphisms (*p*<5e-08) and tested linkage disequilibrium (r^2 =^ 0.001, kb=10000) as instrumental variables, eliminating single nucleotide polymorphisms in linkage disequilibrium to ensure the independence of the instrumental variables (13). With these instrumental variables, we used two-sample Mendelian randomization analysis to analyse the outcome GWAS data and selected eQTLs with causal relationships with mania. By employing two-sample Mendelian randomization, researchers can separately estimate the effects of genetic variations on exposure data and on outcome data. This approach allows them to bypass many of the confounding issues present in traditional observational studies, thereby providing a more accurate estimation of the causal relationship between two variables. The testing results for causal relationships mainly rely on the Wald ratio (WR) and inverse variance weighted (IVW) methods.

### 2.3 Colocalization analysis

We used the coloc package (14) to colocalize all SNPs of genes with causal relationships with outcomes and confirmed the colocalized SNP sites in the gene’s position range by searching the NCBI database. There are four scenarios for colocalization analysis. H0 shows that eQTLs and mania are not significantly related to all SNP sites in the genomic region. The second scenario, H1/H2, shows that eQTLs and mania are significantly associated with SNP sites in the genomic region. H3 shows a significant correlation between eQTLs and mania and SNP sites in the genomic region, but they are driven by different causal mutation sites. H4 shows that eQTLs and mania are significantly associated with SNP sites in the genomic region and are driven by the same causal mutation site (15). Colocalization analysis serves as an effective means of verifying the causal relationship between a gene and its outcome. The causal variable site that the gene shares with mania can be identified, and the genes that successfully colocalized with each other site can be considered core genes.

### 2.4 Enrichment analysis

We used the clusterProfiler package (16) to perform Kyoto Encyclopedia of Genes and Genomes (KEGG) enrichment analysis of the core genes and the GeneMANIA database (17) to analyse the interaction networks among RNA, proteins, DNA and compounds using the walktrap.community algorithm (18) for network modularization analysis and biological enrichment analysis in terms of modules to further reveal the biological processes in which the core genes are involved.

### 2.5 Immune-related analysis

We used the CIBERSORT algorithm (19) to calculate the relative amount of immune cells in the chip expression spectra and then analysed the changes in immune cells between the period of mania onset and remission, as well as the correlation between the core genes and the infiltration of immune cells.

### 2.6 Single-gene GSVA

After the control group samples were removed, the samples were divided into two groups according to the median expression level of the core genes associated with mania, high and low expression. The GSVA algorithm (20) was subsequently used to score the degree of pathway activation in the samples and analyse the differences in pathway activation between the two groups.

### 2.7 Causal relationships between key substances and mania

Consolidating the GSVA results, we analysed substances that have a potential causal relationship with mania using Mendelian randomization. We selected significant and independent SNPs for each key substance as instrumental variables through the IEU database, and we selected genome-wide significant single nucleotide polymorphisms (*p*<5e-06) and tested linkage disequilibrium (r^2 =^ 0.001, kb=10000) as instrumental variables, eliminating the single nucleotide polymorphisms in linkage disequilibrium to ensure the independence of the instrumental variables (13). Moreover, we used Pearson’s algorithm to analyse the correlation between the core genes and the pathway scores of the key substances.

## 3 Results

### 3.1 Genes causally related to mania

**Figure 2:**
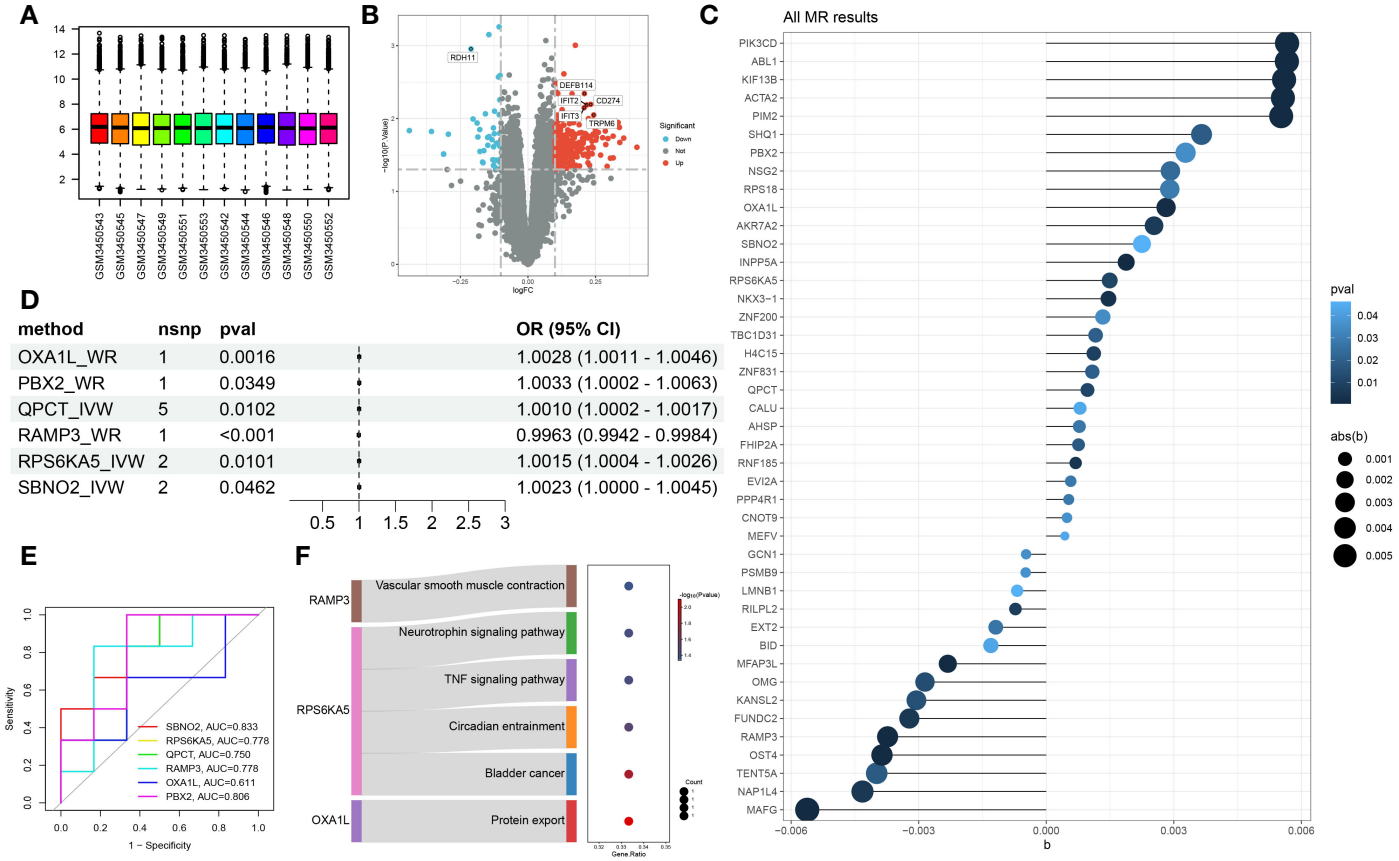
Identification of hub genes associated with manic episodes **(A)** Box plot of sample information in the GSE121963 dataset. **(B)** Volcano diagram of DEGs in the GSE121963 dataset. **(C)** Mendelian randomization results of eQTLs and manic episodes. **(D)** Mendelian randomization results of hub genes and manic episodes after coloc. **(E)** ROC curve of hub genes in manic episodes. **(F)** KEGG enrichment analysis of hub genes.

The GES121963 dataset includes 12 samples: 6 from manic episodes and 6 from periods of remission. The original expression boxplot is shown in **Figure 2A**. After the limma-zoom normalization and differential analysis workflow, a total of 1472 significantly differentially expressed genes (*p*<0.05) were identified, as shown in the volcano plot in **Figure 2B**. Next, the symbols were converted into Ensembl IDs using the org.Hs.eg.db package, and corresponding eQTLs were gathered as exposure data from the eQTLGen database. The outcome data were sourced from the UKB database with the code ukb-b-6906. Mendelian randomization analysis of the two samples identified 46 genes with a causal relationship with manic episodes, as shown in **Figure 2C**.

### 3.2 Colocalization analysis

To rigorously confirm the causal relationship between genes and outcomes, we used coloc to carry out a colocalization analysis of full-length gene SNPs and outcomes, screening for GWAS signals and eQTL loci at the same site with a posterior probability of SNP.PP.H4>0.99. Ultimately, we checked the location of the genes at the National Center for Biotechnology Information, verifying whether the colocalized SNPs were within the range of the genes. This led to the colocalization of 6 SNPs within 6 core genes, as detailed in **Table 2**. The results of two-sample analysis of the 6 core genes and outcomes are shown in **Figure 2D**. As the graph shows, all 6 core genes had a causal relationship with mania, among which RAMP3 was negatively related to manic episodes, while all the other genes were positively related.

**Table 2:** The results of the colocalization analysis.

Symbol	SNPPP.H4	SNP
SBNO2	0.9994316	rs3826958
RPS6KA5	1	rs8016343
QPCT	1	rs7593932
RAMP3	1	rs1294857
OXA1L	0.9961063	rs11157756
PBX2	1	rs576890

### 3.3 Diagnostic performance of core genes and enrichment analysis

We analysed the diagnostic performance of the 6 core genes using the GES121963 dataset as a background, as shown in **Figure 2E**. SBNO2 and PBX2 had excellent diagnostic capabilities and high clinical diagnostic value and warrant further validation and analysis. Through KEGG enrichment analysis of the core genes, we discovered that only 3 genes were enriched in significant signalling pathways, as shown in **Figure 2F**.

**Figure 3:**
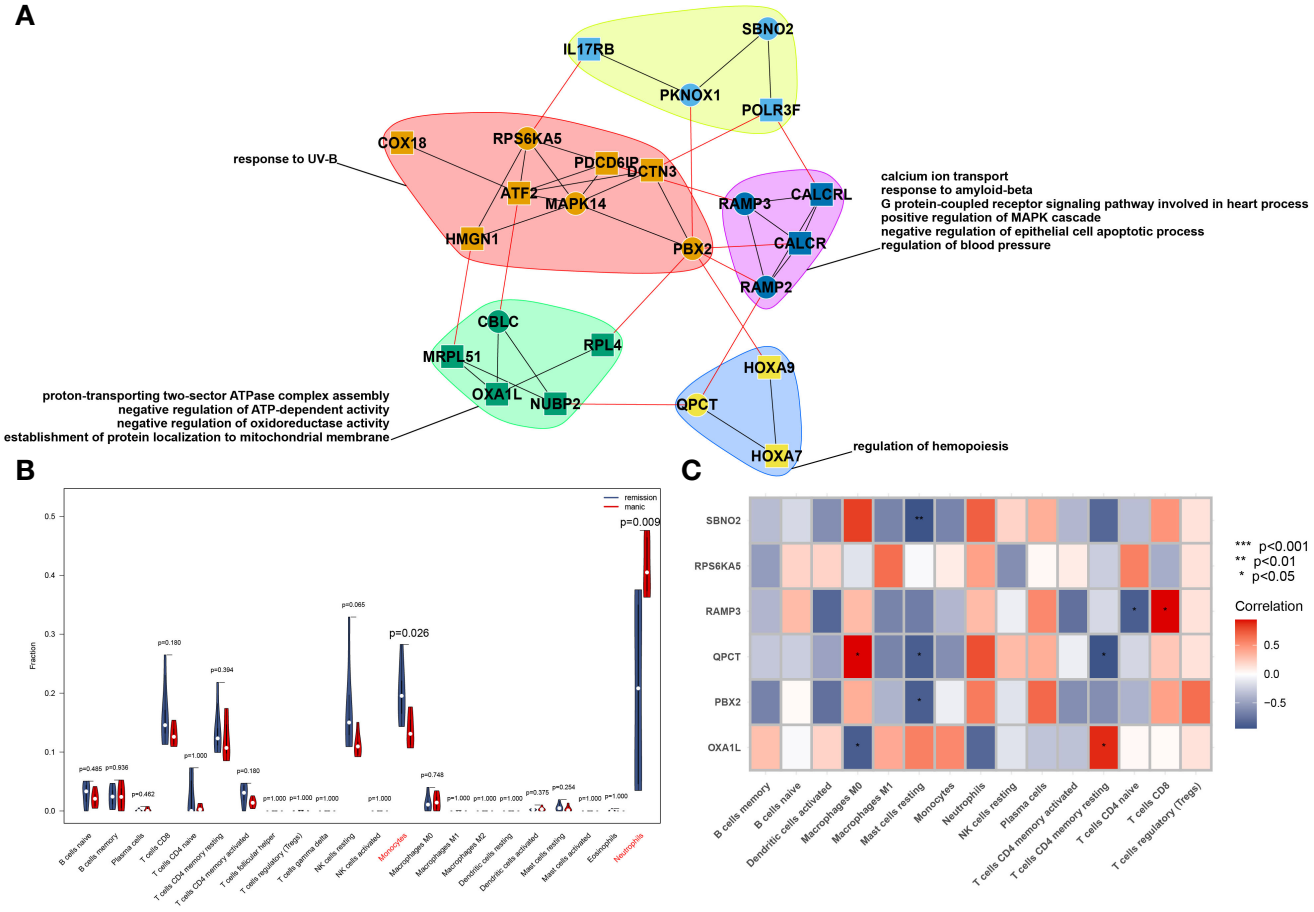
PPI network constructed from GeneMANIA of the hub genes and modular biological process enrichment **(A)**. Calculation of the relative cell content **(B)** and correlation with hub genes **(C)**.

To further study the possible mechanisms of action of the core genes, we used the GeneMANIA database to construct an interaction network and enriched the biological process of each module after module analysis, as shown in **Figure 3A**. OXA1L might influence the occurrence and progression of mania through biological processes such as energy-related activity. Unfortunately, neither SBNO2 nor PBX2 were significantly related to channels or biological processes according to either of the two enrichment methods. We further explored the potential mechanism of genes in the occurrence and development of diseases through single-gene GSVA. In line with the pathway enrichment results, the module in which RAMP3 was located was enriched for the biological process regulating blood pressure.

### 3.4 Immune infiltration analysis

We used the CIBERSORT algorithm to analyse the difference in immune cells between patients with mania during the episodes and remission periods, as shown in **Figure 3B**. According to the calculation results, neutrophil counts significantly increase during manic episodes, while monocyte counts significantly decrease.

On the other hand, we analysed the correlation between core genes and the number of immune cells. The results showed that the six genes were not significantly correlated with neutrophils or monocytes but were significantly correlated with macrophages, mast cells, and T cells, as shown in **Figure 3C**.

### 3.5 Single-gene GSVA enrichment analysis

**Figure 4:**
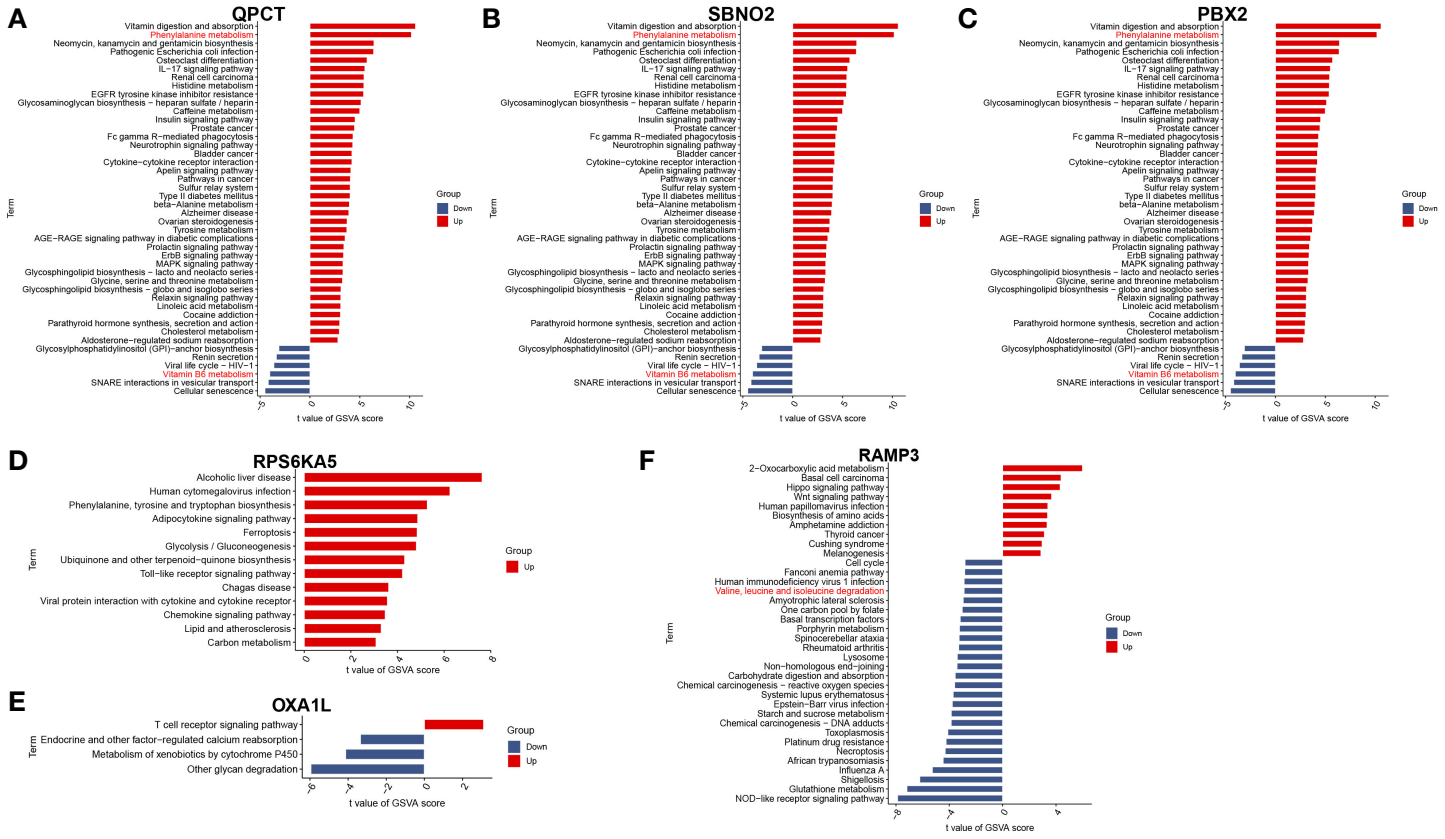
GSVA of hub genes. **(A)** QPCT. **(B)** SBNO2. **(C)** PBX2. **(D)** RPS6KA5. **(E)** OXA1L. **(F)** RAMP3.

To further understand the impact of core genes on this disease, GSVA analysis of a single gene was performed to help us discover signalling pathways that may influence the genes, as shown in **Figures 4A–F**. We are delighted to discover that the GSVA results of the three core genes are completely consistent. This finding suggested that the results may be somewhat skewed due to the sample size but also indicated that the four pathways significantly related to the occurrence and progression of the disease. These four pathways are phenylalanine metabolism, glycosphingolipid biosynthesis-lacto and neolacto series, glycosylphosphatidylinositol (GPI)-anchor biosynthesis, and vitamin B6 metabolism.

According to the single-gene GSVA of RAMP3, six pathways, most of which are disease-related and involve genetic information replication and repair pathways, exhibited significant differences. This suggests that mania can coexist with other diseases or due to the disruption of genetic information, but there are currently no relevant studies to help confirm this phenomenon. Among these different pathways, the most important are the valine, leucine, and isoleucine degradation pathways. This pathway and the phenylalanine metabolism pathway are both related to amino acid metabolism pathways.

### 3.6 Causal relationships between key substances and mania

**Figure 5:**
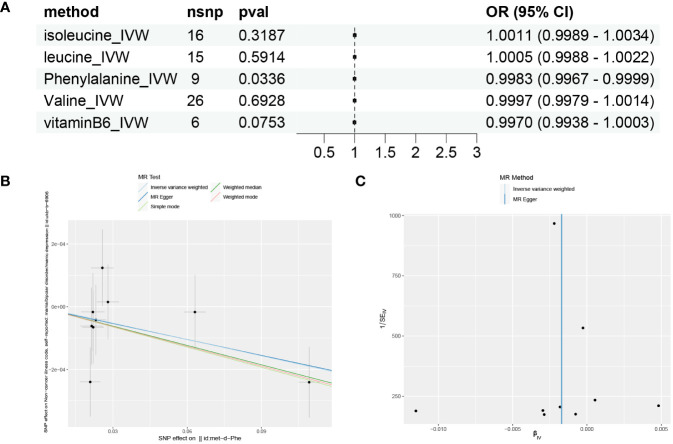
Mendelian randomization results for substances and mania. **(A)** Forest plot of the Mendelian randomization results for five substances. **(B)** Scatter plot of Mendelian randomization results for phenylalanine. **(C)** Funnel plot of Mendelian randomization results for phenylalanine.

Based on the results of GSVA and correlation analysis combined with the literature, we speculate that there are 5 key substances involved in the development of mania: phenylalanine, vitamin B6, valine, leucine, and isoleucine. We then used Mendelian randomization to verify that only serum phenylalanine had a causal relationship with mania, with an OR of 0.9983, suggesting that serum phenylalanine is a protective factor against mania, but the association with mania was relatively low, as shown in **Figures 5A, B**. The scatter plot shows that the regression line obtained for the risk of mania by phenylalanine was basically consistent, indicating reliable results.

MR-Egger intercept analysis did not detect any potential horizontal pleiotropy (*P*=0.9651), indicating that the instrumental variables do not affect the outcome through pathways other than exposure. The Cochran’s Q heterogeneity analysis showed P values all above 0.05, indicating a lack of heterogeneity, as shown in **Table 3**. This is also supported by the funnel plot in **Figure 5C**, which displays a virtually symmetrical causal effect distribution that is not biased by potential factors.

**Table 3:** Cochran’s Q heterogeneity analysis.

id.exposure	id.outcome	method	Q	Q_df	Q_pval
**met-d-Phe**	ukb-b-6906	MR Egger	6.5368	7	0.4786
**met-d-Phe**	ukb-b-6906	Inverse variance weighted	6.5388	8	0.5871

To further illustrate that there is a unidirectional causal relationship between phenylalanine and mania, we conducted a reverse Mendelian randomization study using mania as the exposure and phenylalanine as the outcome. The results suggest that there is a unidirectional causal relationship between phenylalanine and mania, as shown in **Table 4**.

**Table 4:** The findings of the inverse Mendelian randomization study of phenylalanine and mania.

method	nsnp	pval	or	or_lci95	or_uci95
**MR Egger**	10	0.6307	7.26E-15	2.58E-70	2.05E+41
**Weighted median**	10	0.5085	9.041289	0.013227	6180.06
**Inverse variance weighted**	10	0.2860	14.44044	0.10697	1949.384
**Simple mode**	10	0.6075	21.65731	0.000261	1800367
**Weighted mode**	10	0.6332	13.48519	0.000443	410529.9

**Figure 6:**
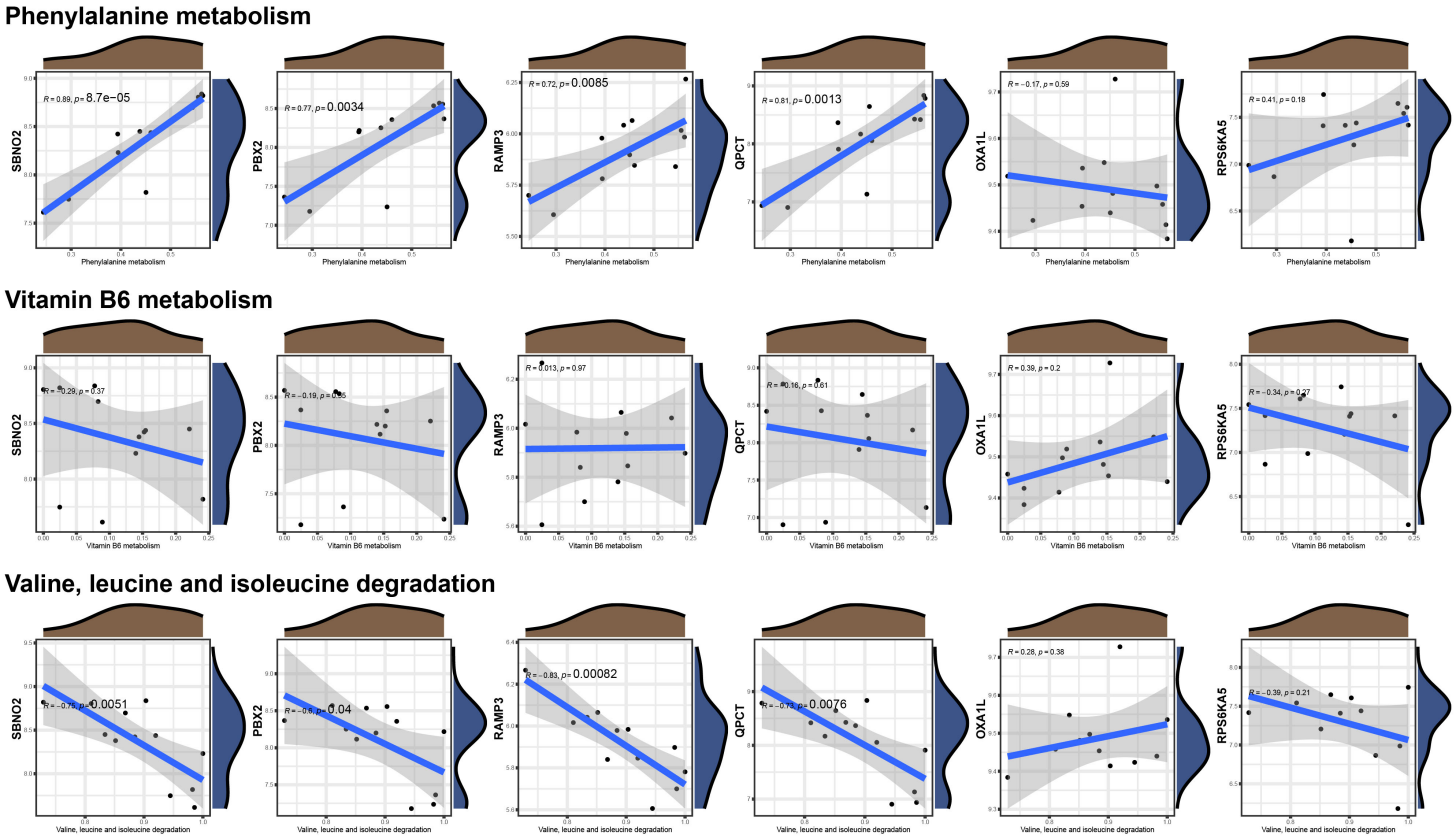
Correlations between the six hub genes and metabolic pathways.

To further verify the correlation between the core genes and key substance-related pathways, we performed a Pearson correlation test, as shown in **Figure 6**. As the results suggest, SBNO2, PBX2, RAMP3, and QPCT are significantly positively correlated with the phenylalanine metabolism pathway and are significantly negatively correlated with the valine, leucine, and isoleucine degradation pathways. None of the six core genes showed a significant correlation with the activation degree of the vitamin B6 metabolism pathway. Interestingly, no pathway showed a difference in the single-gene GSVA analysis grouped based on the expression of OXA1L or RPS6KA5.

## 4 Discussion

Investigating the causal relationship between gene expression and disease is crucial for disease treatment. With the rapid development of genetic and bioinformatics technology, the accuracy of genetic variation measurements is continually improving. This approach significantly reduces the bias that measurement errors may cause in research, hence promoting the widespread use of MR in medical research (21). Therefore, by selecting single nucleotide polymorphisms related to diseases from GWAS data as instrumental variables to replace exposure factors for evaluating the association between exposure and outcome, MR can avert the influence of potential confounding factors on the accuracy of the association results due to its immunity to environmental influences and large sample size. This makes association results even more reliable than observational studies and even randomized controlled trials. This study, for the first time, carried out to determine the causal link between gene expression and mania and identified six core genes with causal relationships through colocalization analyses. Coupled with bioinformatics analysis, this study revealed the potential regulatory mechanisms of core genes and ultimately of phenylalanine with unidirectional causal relationships through further MR analysis. It was also found that four genes, SBNO2, PBX2, RAMP3, and QPCT, are significantly related to the phenylalanine metabolism pathway.

RAMP3 is involved in the vascular smooth muscle contraction pathway, which is intrinsically linked to blood pressure regulation. Blood pressure, an essential indicator of metabolic syndrome, is more likely to indicate anxiety and agitation in patients with metabolic syndrome and depression according to clinical studies (22). On the other hand, research (23) has indicated that the expression of the brain-derived neurotrophic factor (BDNF) gene is significantly decreased in patients during manic episodes and that the expression of this gene is negatively correlated with the severity of manic episodes. In the present study, the neurotrophin signalling pathway, which is regulated by RPS6KA5, was found to be the precise signalling pathway through which BDNF is located. Combined with the positive causal relationship mentioned earlier, these findings indicate that RPS6KA5 is the principal cause of the decrease in BDNF expression, which is worthy of further research and verification.

Moreover, the Circadian entrainment caused by RPS6KA5 has been proven to be connected with manic episodes. The melatonin levels of patients during manic episodes are significantly greater than those of healthy controls and patients with depressive episodes during the day (24). The finding that the TNF signalling pathway is regulated by RPS6KA5 is consistent with the findings of existing research. Research shows that peripheral blood TNF-α levels are significantly greater in patients with a normal mood and in healthy individuals during manic episodes than in healthy individuals (25). Interestingly, we found calcium ion transport in our enrichment results, and calcium ions are a critical part of blood pressure regulation (26). Blood pressure regulation is closely related to biological clocks. Biological clock disorders have been observed in schizophrenia and many other mental illnesses. These diseases are accompanied by a decrease in amplitude or phase changes in various rhythms, including sleep-wake rhythms, blood pressure, hormone rhythms (cortisol and melatonin), and 24-hour circadian gene expression. At a broader epidemiological level, people are more prone to depressive symptoms in the short daylight season of winter and more likely to exhibit manic symptoms in the long daylight season of summer, showing seasonal variations. At the molecular mechanism level, animal model studies not only reveal the potential metabolic and immune pathways that may establish a connection between the biological clock and mood regulation but also reveal specific molecules, cells, and physiological pathways. The biological clock gene directly regulates tyrosine hydroxylase and monoamine oxidase A, which are the rate-limiting enzymes in dopamine generation and degradation processes. Given the significant role of dopamine in schizophrenia and many other mental illnesses, understanding the regulatory mechanism of the biological clock may lead to new treatment approaches (27).

In earlier studies, it was shown that the number of neutrophils in manic patients is significantly greater than that in patients with schizophrenia. In recent years, the neutrophil-lymphocyte ratio has been developed as a diagnostic marker for manic episodes (28), and the monocyte-lymphocyte ratio is significantly greater than that in nonmanic periods, which differs from our research results. It has been found that peripheral blood macrophage factors are significantly more common in manic patients than in mentally stable patients and healthy individuals (25). On the other hand, through diffusion tensor imaging (DTI), the team of Paola Magioncalda depicted the distribution of T cells in the white matter region of the brain in manic patients and emotionally stable patients and found that, in manic patients, CD4+ T cells significantly increased, while CD8+ T cells and their subgroups decreased significantly (29). Although the CIBERSORT algorithm used in this study did not detect a difference in T cells, which may be due to sample differences, in this study, three genes that were significantly correlated with T cells and had causal relationships with mania, RAMP3, QPCT, and OXA1L, were screened, providing a reference for further study of the connection between T cells and mania.

Two phospholipid pathways have not been shown to be related to mania. However, glycosphingolipid (GSL) and glycosylphosphatidylinositol (GPI) are the two major glycolipids expressed by eukaryotic cells, and their metabolisms share the same machinery (30). Research indicates that GSL and GPI are the material basis for neuronal metabolism and that abnormal GSL metabolism seriously damages mitochondrial function, and pathological changes related to mental illness result from decreased neurotransmitter release and transmission (31). This result provides a new direction for future research on mania.

Relevant clinical studies have confirmed that vitamin B6 adjuvant therapy can effectively relieve depression and anxiety in patients with mental illnesses (32), and in the present study, the vitamin B6 metabolism pathway was significantly different, suggesting that the content of vitamin B6 may be key for improving mania. A cross-sectional study revealed that not only the content of vitamin B6 but also the content of minerals such as magnesium and zinc are significantly correlated with mania (33). Simultaneously, another RCT study on vitamin B6 (34) showed that after controlling for confounding factors, oral vitamin B6 could not improve the mental health of manic patients. Considering the different conclusions on vitamin B6, we will use the method of Mendelian randomization in the next step to analyse whether there is a causal relationship between vitamin B6 and mania.

A few studies have confirmed that amino acids are closely related to the occurrence and development of mania, and phenylalanine and leucine are closely related or have a causal relationship. Casein glycomacropeptide (CGMP) is a peptide lacking phenylalanine, tyrosine, and tryptophan that can rapidly change the levels of amino acids and monoamines in the brain and periphery (35). A clinical study of humans by Erik Roj Larsen (36) revealed that, compared with the 20 g and 40 g doses, 60 g of CGMP was well tolerated, and after 3-4 hours of medication, it resulted in the maximum depletion of phenylalanine and tyrosine. Phenylalanine and tyrosine are essential components of dopamine synthesis in the brain, so CGMPs can significantly reduce the synthesis of dopamine. According to previous research, leucine can enhance the effect of CGMP-induced phenylalanine depletion (35).

However, the finding that phenylalanine is a protective factor contradicts the findings of existing clinical research, which states that phenylalanine depletion reduces the biosynthesis of dopamine, thereby alleviating manic-like behaviour. This phenomenon also shows that additional in-depth research is required into the mechanism of action of phenylalanine. A study (35) revealed that leucine can enhance the effect of phenylalanine depletion on CGMPs, but this study did not find a causal relationship between leucine and mania. We can infer that phenylalanine depletion is not the main reason for the existing relationship between phenylalanine and mania. If phenylalanine depletion is the major reason for the causal relationship between phenylalanine and mania, then leucine might be able to have a causal relationship with mania by enhancing the efficiency of phenylalanine depletion.

This study helps to screen four key drug target genes for mania and furthers the understanding of the potential regulatory mechanisms of these four genes. This study has several limitations. There was some degree of heterogeneity in the analyses. Due to the use of GWAS data, it is impossible to explore any potential nonlinear relationships or stratified effects that vary due to age, health status, or sex, which might lead to heterogeneity. While the screening of drug target genes shows great potential in the treatment of mania, specific treatment regimens need further research and clinical practice for confirmation. The consolidated GWAS data include only individuals of European descent; the conclusions may not fully represent the entire population. Future studies should be conducted to validate the applicability of these results to other races.

In summary, from a genetic perspective, the results of this study reveal a causal link between six genes and mania. Bioinformatics and MR integrated analysis established the causal relationship of the key substance in the pathway, phenylalanine, with mania. Ultimately, four genes significantly correlated with the key pathway, phenylalanine metabolism, were identified as drug target genes.

## Data availability statement

The original contributions presented in the study are included in the article/supplementary material. Further inquiries can be directed to the corresponding author.

## Author contributions

F-BX: Formal analysis, Writing – original draft, Writing – review & editing. SH: Writing – review & editing. J-JW: Data curation, Writing – review & editing. X-ZW: Conceptualization, Funding acquisition, Investigation, Writing – review & editing.
